# ﻿Four new species of *Leuctra* Stephens, 1836 from the Balkans (Plecoptera, Leuctridae)

**DOI:** 10.3897/zookeys.1218.120744

**Published:** 2024-11-15

**Authors:** Dávid Murányi, Tibor Kovács

**Affiliations:** 1 Department of Zoology, Eszterházy Károly Catholic University, Leányka út 6, Eger H-3300, Hungary Eszterházy Károly Catholic University Eger Hungary; 2 Mátra Museum of the Hungarian Natural History Museum, Kossuth Lajos u. 40, Gyöngyös H-3200, Hungary Mátra Museum of the Hungarian Natural History Museum Gyöngyös Hungary

**Keywords:** Albania, Bosnia & Herzegovina, *Leuctraenigma*, *L.golija*, *L.puskasi*, *L.visitor*, Montenegro, Serbia

## Abstract

Four, presumably microendemic new *Leuctra* species are described on the basis of morphology of the adult males and females. Each species was collected only in a single mountain range of the western Balkans: *Leuctraenigma* Kovács & Murányi, **sp. nov.** (Albania, Çermenikë), *L.golija***sp. nov.** (Serbia, Golija), *L.puskasi***sp. nov.** (Bosnia & Herzegovina, Kozara), *L.visitor***sp. nov.** (Montenegro, Visitor). Their morphological affinities and ecology are discussed, phylogenetic relations will be described in the framework of ongoing molecular studies of Balkan *Leuctra* species. The occurrences of the new species are depicted on a map. A list of Balkan endemic *Leuctra* species is given. *Leuctradalmoni* Vinçon & Murányi, 2007 is new for Albania and Montenegro, *Nemourauncinata* Despax, 1934, *N.marginata* Pictet, 1836 and *Isoperlaburesi* Raušer, 1962 are new for Albania, *Nemourasciurus* Aubert, 1949 is new for Bosnia & Herzegovina, while Capnias. l.viduarilensis Raušer, 1962 and *L.metsovonica* Aubert, 1956 are new for Montenegro.

## ﻿Introduction

The Balkan Peninsula is one of the hot spots of West Palaearctic Plecoptera diversity, with more than 200 species reported from the area to date (Graf et al. 2009; DeWalt et al. 2023). However, exploration of the region is far from complete (Sánchez-Campaña et al. 2023), and our knowledge on the fauna of different countries rather unbalanced (Kaćanski 1979; Popijač and Sivec 2009b; Murányi et al. 2014b; Petrović et al. 2014; Karaouzas et al. 2016; Pešić et al. 2018; Tyufekchieva et al. 2019; Bilalli et al. 2020; Murányi and Kovács 2024) According to the latest checklist (Murányi 2009a), the Balkans harbours 197 species, of which 84 have wider distribution, while 113 species are endemic or subendemic to the peninsula. Since then, twelve new species and a subspecies were described from the Balkans (Murányi 2009b, 2011; Graf and Bálint 2010; Graf et al. 2012, 2018; Kovács et al. 2012; Murányi et al. 2014a; Vinçon 2015; Hlebec et al. 2021; Reding et al. 2023), and eleven species were reported first from the peninsula (Popijač and Sivec 2009a; Petrović et al. 2014; Kazanci 2015).

**Figure 1.**
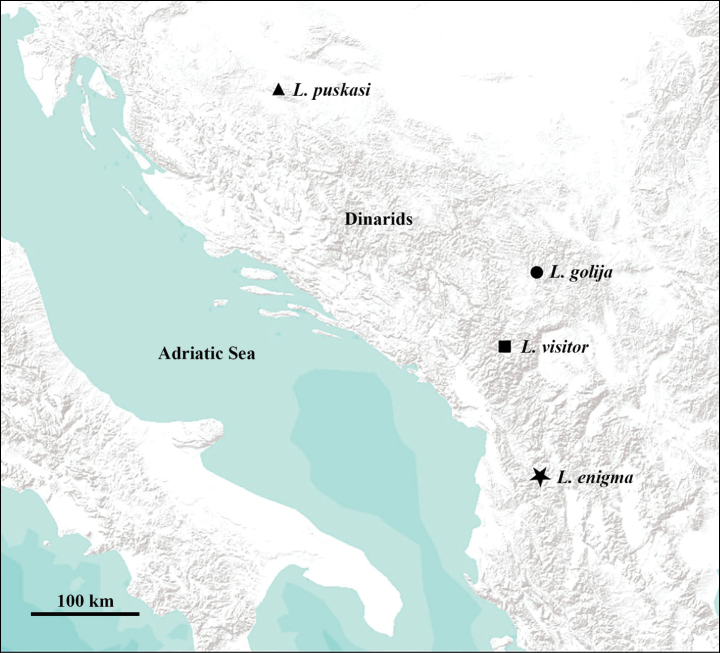
Collection sites of the four new *Leuctra* species.

During the last twenty years, we conducted several collecting trips to all Balkan countries. Some of the results, mostly new species descriptions, were published separately but the bulk of the faunistic data and several undescribed species still remained unpublished. We faced several complex taxonomic problems that requires an integrative approach (e.g., Murányi et al. 2016), and so most species descriptions were postponed. While molecular studies are underway, herein we give the morphological description of four distinctive new *Leuctra* Stephens, 1836, three of which were collected in the Dinaric, and one in the Western-Central Balkans (Fig. 1).

## ﻿Materials and methods

Specimens were collected by hand or by beating sheet and stored in vials with 70% ethanol. Further specimens for molecular studies were transferred in 96% ethanol. Holotypes and most paratypes are deposited in the Collection of Smaller Insect Orders, Department of Zoology, Hungarian Natural History Museum, Budapest, Hungary (**HNHM**) and in the Mátra Museum of the Hungarian Natural History Museum, Gyöngyös, Hungary (**MM**), further paratypes are deposited in the Monte L. Bean Life Science Museum, Brigham Young University, Provo, Utah, US (**BYU**), Gilles Vinçon Collection, Grenoble, France (**CGV**), Department of Zoology, Eszterházy Károly Catholic University, Eger, Hungary (**EKCU**) and in the Stuttgart State Museum of Natural History, Stuttgart, Germany (**SMNS**).

Drawings were made with the aid of a drawing tube applied on a Nikon SMZ1500 and a Nikon SMZ10 microscopes. Terminology of the species description follows Murányi (2007) and Vinçon and Murányi (2007).

Distribution and ecology of the Balkan endemic *Leuctra* were compiled from the following literature (in addition to the original descriptions): Braasch and Joost (1971), Darilmaz et al. (2016), Ikonomov (1986), Kaćanski (1975), Kovács and Murányi (2023), Murányi (2011), Murányi et al. (2014b, 2016), Pešić et al. (2018), Petrović et al. (2014), Ravizza (2002a), Sivec (1980), and Tierno de Figueroa and Fochetti (2001).

## ﻿Taxonomic account

### ﻿ Leuctra enigma

Taxon classificationAnimaliaPlecopteraLeuctridae

Kovács & Murányi sp. nov.

6EAE8429-DA2E-58F4-8A81-3DD7F30E156A

https://zoobank.org/F0FF7DC4-7EA8-4BDE-9042-34D956A63609

**Figure 2.**
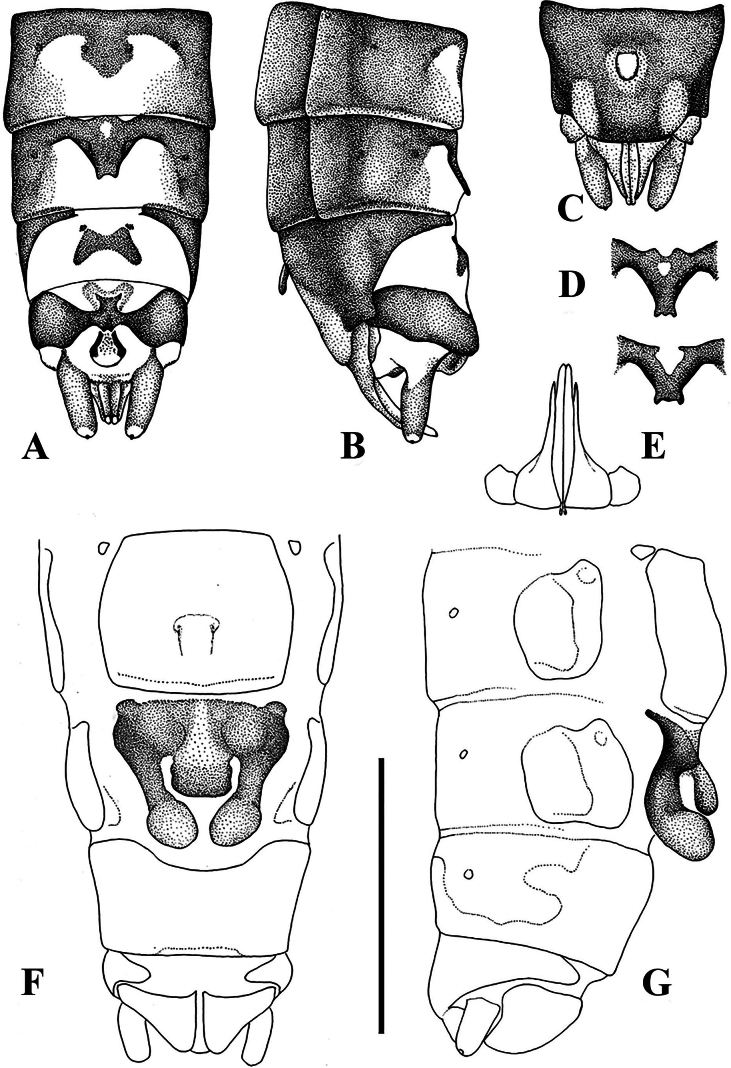
Terminalia of *Leuctraenigma* sp. nov. with setation omitted **A** male terminalia, dorsal view **B** same, lateral view **C** same, ventral view **D** variations of male tergite 8, dorsal view **E** male paraprocts and specillae, caudal view **F** female terminalia, ventral view **G** same, lateral view. Scale bar: 1 mm.

**Figure 3.**
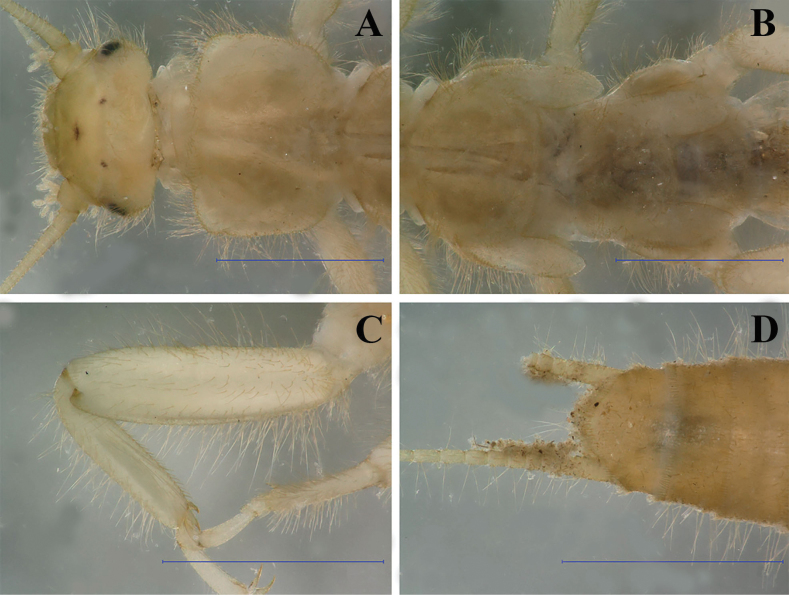
Putative larva of *Leuctraenigma* sp. nov. **A** head and pronotum, dorsal view **B** meso- and metathorax, dorsal view **C** hind leg, dorsal view **D** terminalia, dorsal view. Scale bars: 1 m.

**Figure 4.**
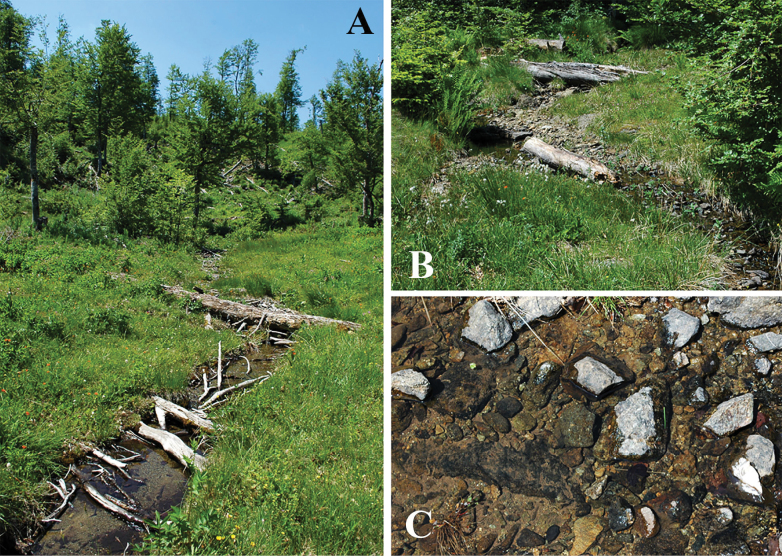
Habitat of *Leuctraenigma* sp. nov. **A** type locality: partly open brook in the Çermenikë Mts. **B** section at forest edge **C** substrate of the type locality.

Figs 1 , 2 , 3 , 4 , 7A, E

#### Type material.

***Holotype*** male: Albania: • Dibër county, Bulqizë municipality, Çermenikë Mts, brooks in open forest beneath Mt. Kaptinë, 1600 m, 41°23.199'N, 20°17.338'E, 10.x.2012 (field number: loc.25), leg. P. Juhász, T. Kovács, D. Murányi, G. Puskás (MM: 2012-178, PLETYP-30). ***Paratypes***: • same locality and date: 1♂ 1♀ (BYU) • 1♂ 1♀ (CGV) • 5♂ 6♀ (HNHM: PLP4184) • 5♂ 10♀ (MM: 2012-178, PLETYP-31) • 1♂ 1♀ (SMNS).

#### Other materials.

• Same locality, 21.vi.2012 (loc.58), leg. Z. Fehér, T. Kovács, D. Murányi: 3 putative larvae (HNHM: PLP4099) • 2 putative larvae (MM: 2012-1) • same locality, 27.v.2013 (loc.19), leg. P. Juhász, T. Kovács, G. Magos, G. Puskás: 2 putative larvae (MM: 2013-19).

#### Diagnosis.

Macropterous in both sexes. Male tergite VII with huge membranous portion; tergite VIII with large, triangular posteromedial process, terminating in bi- or trilobed tip; tergite IX with large, lobed posteromedial sclerite supported by anterior sclerotised spots; tergite X posterior margin with deep and wide notch; sternite IX bears a vesicle shorter than 1/5 segment length; paraproct tip pointed, specillum slightly longer than paraproct, tip blunt. Female terga I–VIII with medial sclerite; subgenital plate with large lobes terminating in swollen and raised apex, central plate with anterolateral swellings and pale medial portion, posterior portion trapezoid in ventral view and nose-shaped in lateral view, well past by the lobes. Larva stout and with long setation, eyes small, clypeus lacks pointed corners.

#### Description.

Medium sized, robust species, both sexes macropterous. Forewing length: holotype 6.0 mm, male paratypes 5.8–6.4 mm, female paratypes 6.5–7.8 mm; body length: holotype 6.5 mm, male paratypes 6.0–6.8 mm, female paratypes 8.0–8.8 mm. Setation generally short and dense. General colour dark brown. Head brown with distinct M-line and occipital rugosities, compound eyes relatively small; antennae dark brown, palpi brown. Pronotum brown to dark brown, wider than long and having rounded corners, rugosities distinct. Legs brown to dark brown, tarsi slightly darker. Wings brownish but hyaline, venation brown.

Male abdomen (Figs 2A–E, 7A): Tergite I membranous posteriorly and medially, with two medial spots; tergite II poorly sclerotised anteriorly. Transverse row of four pigmented spots distinct on terga II–VIII. Terga II–III with poorly developed antecosta, terga IV–VII with entire antecosta, terga III–VI full sclerotised. Tergite VII with huge medial membranous portion, a rounded or arrow-shaped sclerotised area extends between the medial pair of pigmented spots. Tergite VIII: antecosta nearly entire, gradually weakened in the medial 1/5, sometimes interrupted by a narrow medial notch; the posteromedial process is triangular, originated in the anterior 1/3 of the segment and reach the posterior 1/3 by its weakly bilobed or trilobed tip, medial portion with small membranous portion, medial pair of pigmented spots recognisable by the lateral sides of the process; the process is not darker than the lateral areas of the segment, wider than 1/3 segment width, slightly erect in side view; lateral edges of the medial membranous area obscure, while the anterior edge is sharply defined. Tergite IX mostly membranous, antecosta interrupted in the medial 1/3 of its length; posteromedial sclerite large, weakly bilobed anteriorly while posterior portion with long, diverging lobes, the sclerite is anteriorly supported by two sclerotised spots. Anterior margin of tergite X bilobed anteriorly but sclerotisation of lobes obscure; posterior margin with deep notch, as wide as 1/2 tergite width; medial portion of the notch with small protrusion at the origin of epiproct. Epiproct drop-shaped, sclerotised only at its sides, stalk gradually widening. Cercus simple, covered with long setae. Sterna II–VIII simple, sternite IX bears a very small, rounded vesicle shorter than 1/5 of segment length; posterolateral portions of the sternum pale coloured and weakly sclerotised. Paraproct with moderately wide base, narrowing after basal 1/3, apex gently curved in lateral view and tapering towards a pointed tip. Base of paraproct connected to a subrectangular lateral expansion. Specillum longer than the paraproct, gently curved in lateral view and ending in a blunt tip.

Female abdomen (Figs 2F–G, 7E): Terga I–VIII with transverse row of four pigmented spots clearly visible; terga I–VIII mostly membranous but with lateral sclerites and a medial sclerite as well; medial sclerite is a longitudinal stripe, more widened on terga II and III; tergite IX mostly, tergite X fully sclerotised. Sterna I–VII simple, sterna II–VII with one subrectangular median sclerite and two small anterior sclerites that are not fused. Subgenital plate of sternite VIII large and complex, not fused with other sclerites, lacks distinct setation. It has two large and well-defined lobes, darker brown than the remainder of the plate; the lobes terminate in a swollen, rounded apex that are paler than the base of the lobes, raised in lateral view and converging in ventral view. The central plate has two anterolateral swellings, medial portion pale and lightly sclerotised; the plate terminates in a trapezoidal bulge, raised and nose-shaped in lateral view, clearly separated from the lateral lobes that extend beyond it. Sternite IX with wide but shallow anterior indentation. Paraproct, cercus, and epiproct simple. Spermathecal sclerite thin, ring-shaped, with small anterior teeth and long posterior teeth.

Putative larva (Fig. 3): Body stout, length of the penultimate or younger larvae 7.0–7.5 mm. General colour pale brown, without distinctive markings, pilosity long and distinct. Head much wider than long, eye small, shorter than the widely rounded occipital region, ocelli distinct, clypeus not modified. Palpi simple, antenna longer than 1/2 body length. Pronotum much wider than long. Wing pads under development, posterior edge of meso- and metathorax between wing pads rounded, not like the typical *prima-hippopus-inermis* group shape. Legs short, tibiae not longer than femora, stretched hind leg reaches back to the seventh abdominal segment. Abdomen stout, integument matt pale brown, paraproct triangular. Cercus nearly as long as abdomen, with 25 cylindrical segments, medial segments twice longer than wide. Setation of the larva: antenna and palpi with short setae but head and clypeus with dense and erect setation, longest setae as long as the distance between hind ocelli; marginal setae continuous on pronotum, as long as the setae of the head; wing pads with similar long, erect and dense setae; femora and tibiae with hairs as long as the width of each femora, distributed on the whole surface but not forming swimming fringe; tarsi with short and scarce setation; abdominal segments evenly setose, with erect setae approximately as long as the setae of the head; cercomeres bald besides the erect apical whorl, slightly shorter than abdominal setae.

#### Affinities.

The new species is an isolated member of the *hippopus* group. The male is rather distinctive, the only *Leuctra* with comparable posteromedial process of tergite VIII is the Caucasian-Anatolian *L.martynovi* Zhiltzova, 1960. However, *L.martynovi* differs by larger posteromedial sclerite of tergite IX, shallow posterior notch of tergite X, and larger vesicle of sternite IX. The subgenital plate of the female shows similarity with the narrowly defined *hippopus* group (*hippopus* subgroup, sensu, e.g., Ravizza 2002b), having large lobes and a pale central plate. However, the combination of bulging lobes of the subgenital plate, central plate with anterolateral swellings and trapezoid, bulging posterior portion, already distinguish it from all other *Leuctra*. The putative larva is distinctive by its stout body and dense, long setation. Among the Balkanian *Leuctra*, only *L.nigra* (Olivier, 1811) and *L.hirsuta* Bogoesco & Tabacaru, 1960 are having similar long setation, however, their body is less stout, and their eyes are larger. The Alpine-Carpathian *L.braueri* Kempny, 1898 has similarly stout and hairy larva, but its clypeus is armed with prominent pointed corners.

#### Distribution and ecology.

The species was collected at a single high elevation site of the Çermenikë Mts, central Albania (Fig. 1). It was not found at any further localities, despite of intensive collecting efforts during the last twenty years conducted in central Albania. The habitat is disturbed by partial deforestation, most sections of the two confluent brooks run open or in bush of young beech (Fig. 4A, B). The brooks run moderately fast, 0.5–1 meter wide and not deeper than 20 centimetres (both in June and October). The substrate is stony, mixed with small sandy patches, few patches of dead wood and partly submerged plants (Fig. 4B, C). The specimens were swarming together with *L.hirsuta* in October 2012. When we visited the locality in late spring and early summer, a more diverse fauna was found: 21.vi.2012 (leg. Z. Fehér, T. Kovács, D. Murányi): adults of *Brachypterahelenica* Aubert, 1956b, *Leuctrahippopoides* Kaćanski & Zwick, 1970, *L.metsovonica* Aubert, 1956, *Nemurellapictetii* (Klapálek, 1900), *Nemouracinereacinerea* (Retzius, 1783), *N.marginata* Pictet, 1836, Perlacf.pallida Guérin-Méneville, 1843, *Isoperlaburesi* Raušer, 1962 and *Siphonoperlaneglecta* (Rostock, 1881); 27.v.2013 (leg. P. Juhász, T. Kovács, G. Magos, G. Puskás): adults of *B.helenica*, *L.hippopoides*, L.cf.dalmoni Vinçon & Murányi, 2007, *Nemouracinereacinerea*, *N.uncinata* Despax, 1934, *N.marginata*, *Nemurellapictetii*, *Siphonoperlaneglecta* and *S.graeca* (Aubert, 1956), and putative larvae of the new species were found. Leuctracf.dalmoni, *Nemourauncinata*, *N.marginata* and *Isoperlaburesi* are new findings for Albania. Records of other aquatic insects collected at the type locality were already published: Ephemeroptera (Kovács and Murányi 2013): *Baetismuticus*, *Habrophlebialauta*; Odonata (Murányi and Kovács 2013): *Cordulegasterbidentata*; Trichoptera (Oláh and Kovács 2012b, 2013): *Agapetusiridipennis*, *Chaetopteryxbosniaca*, *Psychomyiapusilla*, *Cyrnustrimaculatus*, *Lepidostomahirtum*, *Drususplicatus*, *Mystacidesazureus*.

#### Etymology.

The name *enigma* (Latin; enigma or riddle) refers to the strange fact that such a distinctive new species was found only at a single locality. Used as a noun, gender neutral.

### ﻿ Leuctra golija sp. nov.

Taxon classificationAnimaliaPlecopteraLeuctridae

42DFF825-01A6-53BD-AAA8-79B5D3C1D0E1

https://zoobank.org/776454AB-1086-4B10-A56B-636E13224914

**Figure 5.**
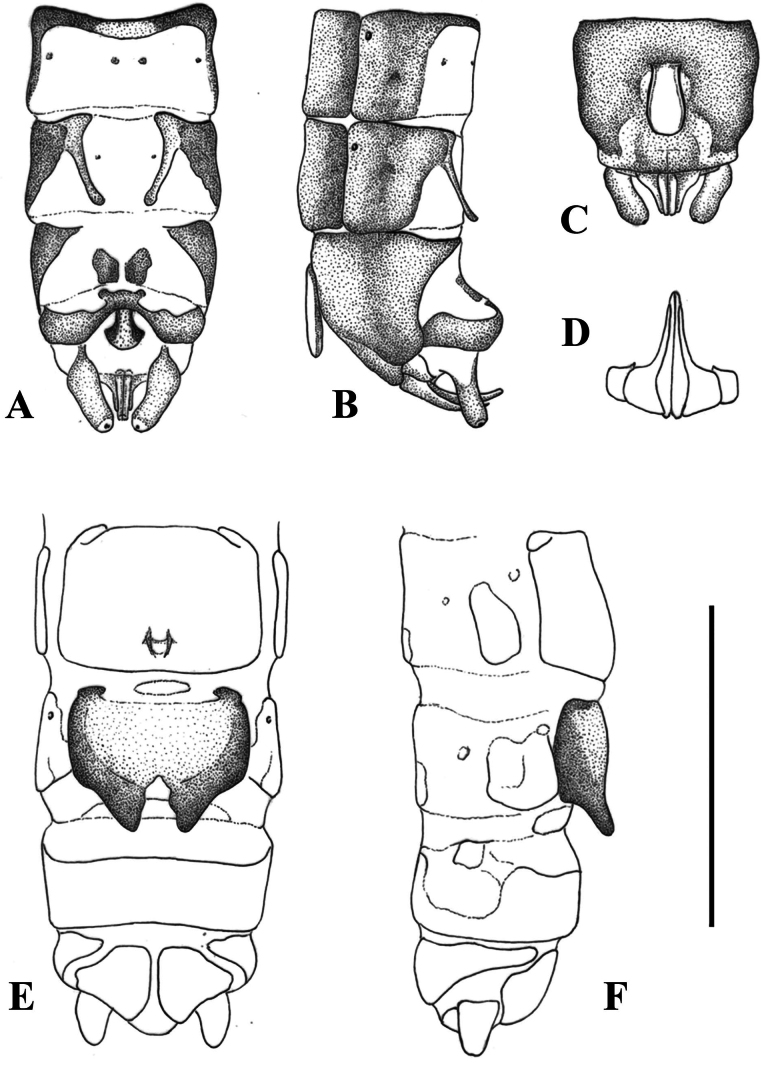
Terminalia of *Leuctragolija* sp. nov. with setation omitted **A** male terminalia, dorsal view **B** same, lateral view **C** same, ventral view **D** male paraprocts and specillae, caudal view **E** female terminalia, ventral view **F** same, lateral view. Scale bar: 1 mm.

**Figure 6.**
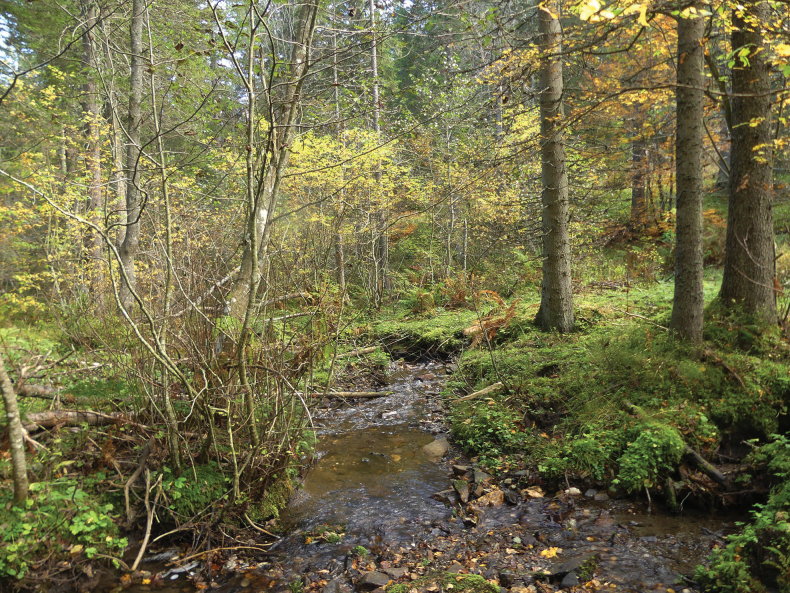
Habitat of *Leuctragolija* sp. nov.: Moravica Stream in the Golija Mts (type locality, 23.x.2023).

**Figure 7.**
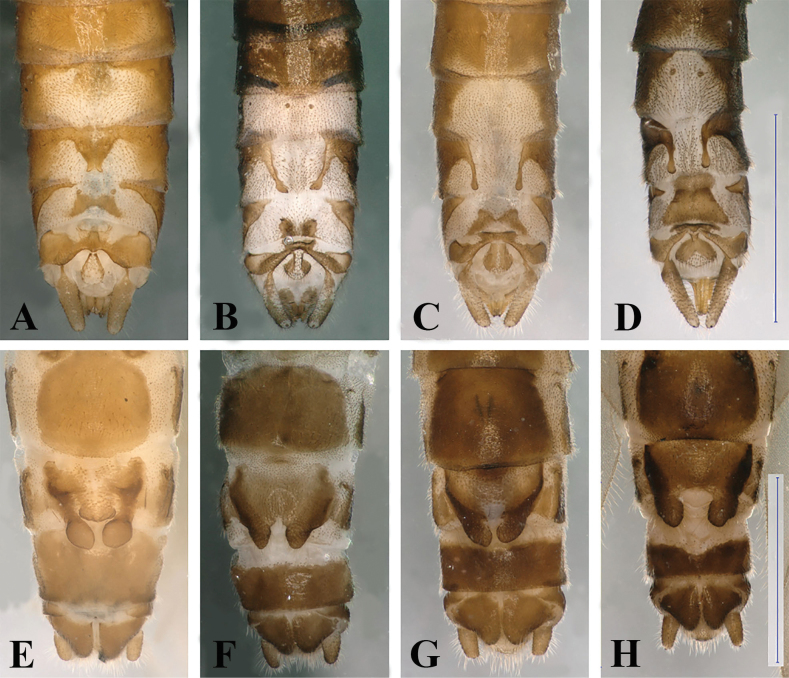
Terminalia of four Balkan members of the *Leuctrahippopus* group **A–D** male terminalia, dorsal view **E–H** female terminalia, ventral view. **A, E***L.enigma* sp. nov., Çermenikë Mts, Albania **B, F***L.golija* sp. nov., Golija Mts, Serbia **C, G***L.pseudohippopus* Raušer, 1965, Stara Planina, Serbia **D, H***L.hippopoides* Kaćanski & Zwick, 1970, Sutjeska, Bosnia & Herzegovina. Scale bars: 1 mm (**D** for **A–D**, **H** for **E–H**).

Figs 1 , 5 , 6 , 7B, F

#### Type material.

***Holotype*** male: Serbia: • Moravica district, Ivanjica municipality, Golija Mts, Dajići, Moravica Stream at Milošica, 1505 m, 43.3384°N, 20.2507°E, 6.v.2023 (field number: loc.16), leg. L.P. Kolcsár, T. Kovács, D. Murányi (MM: 2023-51, PLETYP-32). ***Paratypes***: • same locality and date: 1♂ 1♀ (BYU) • 1♂ 1♀ (CGV) • 14♂ 6♀ (EKCU: PLP5721) • 10♂ 3♀ (MM: 2023-51, PLETYP-33) • 1♂ 1♀ (SMNS) • same locality, 26.vi.2018 (loc.1), leg. P. Juhász, T. Kovács, D. Murányi: 1♀ (HNHM: PLP5003) • 1♂ (MM: 2018-43, PLETYP-34) • Raška district, Novi Pazar municipality, Golija Mts, Radaljica, spring brooks in forest edge, 1575 m, 43.2743°N, 20.3467°E, 6.v.2023 (loc.17), leg. L.P. Kolcsár, T. Kovács, D. Murányi: 1♂ (EKCU: PLP5727) • 2♂ 1♀ (MM: 2023-52, PLETYP-35).

#### Diagnosis.

Macropterous in both sexes. Male tergite VII mostly membranous, with bicoloured antecosta; tergite VIII with converging pair of slender, pale brown processes, medial membranous area rounded between the processes, triangular between the processes and the lateral sclerotised portions; tergite IX with small posteromedial sclerite, divided into leaf-like portions; tergite X posterior margin with wide and deep notch; epiproct sclerotised only at its sides, stalk nearly as long as the rounded apex; sternite IX bears a vesicle 1/2 as long as segment length; specillum slightly longer than paraproct, tip of paraproct acute, tip of specillum blunt. Female subgenital plate large and bicoloured, lobes large, triangular, and not converging, slightly raised in lateral view and the notch between them is triangular; spermathecal sclerite ring-shaped, with large and converging posterior teeth.

#### Description.

Medium sized, slender species, both sexes macropterous. Forewing length: holotype 5.6 mm, male paratypes 5.4–6.0 mm, female paratypes 6.6–7.0 mm; body length: holotype 5.4 mm, male paratypes 5.2–6.4 mm, female paratypes 5.5–7.2 mm. Setation generally short and dense. General colour brown to dark brown. Head and antennae dark brown, palpi brown. Pronotum brown to dark brown, as wide as long or slightly wider, having rounded corners, rugosities distinct. Legs brown to dark brown. Wings brownish but hyaline, venation brown.

Male abdomen (Figs 5A–D, 7B): Tergite I membranous medially, with two medial spots; terga II and III poorly sclerotised anteriorly. Transverse row of four pigmented spots distinct on terga II–VII. Terga II–III with widely divided antecosta, terga IV–VII with entire antecosta, terga IV–VI full sclerotised. Tergite VII mostly membranous, sclerotised only laterally and in the widened antecosta that is distinctly bicoloured: dark brown laterally and pale brown medially. Tergite VIII: antecosta medially divided nearly in the 1/2 width of the segment; paired processes originated from the end of antecosta and reaches the posterior end of the segment, pale brown coloured, converging posteriorly, very slender, and only slightly widened apically, raised in lateral view; the medial membranous area well limited, rounded between the processes and with two sclerotised spots, triangular between the processes and the lateral sclerotised portion of the segment. Tergite IX mostly membranous, antecosta interrupted in the medial 1/2 of its length; posteromedial sclerite small, divided into leaf-like portions. Anterior margin of tergite X bilobed anteriorly; posterior margin with deep and wide notch, as wide as 1/2 tergite width. Epiproct large, sclerotised only at its sides, stalk nearly as long as the rounded apex. Cercus simple, covered with long setae. Sterna II–VIII simple. Sternite IX bears a long, tongue-shaped vesicle 1/2 as long as segment length; posterolateral portions of the sternum pale coloured and weakly sclerotised, connected to pale area around the base of vesicle. Paraproct with moderately wide base, narrowing after basal 1/3, apex gently curved in lateral view and tapering towards an acute tip. Base of paraproct connected to a subrectangular lateral expansion, having a small inner apical tip. Specillum longer than the paraproct, gently curved in lateral view and ending in a blunt tip.

Female abdomen (Figs 5E, F, 7F): Terga I–VIII with transverse row of four pigmented spots clearly visible; terga I–VIII mostly membranous but with lateral sclerites; small medial sclerite is present on terga VII–VIII, sometimes also on tergite II; tergite IX mostly, tergite X fully sclerotised. Sterna I–VII simple, sterna II–VII with one subrectangular median sclerite and two small anterior sclerites that are fused with median sclerite only on sternite VII. Subgenital plate of sternite VIII large, setation not distinctive; not fused with lateral sclerites but touching with, a small sclerotised portion is visible on the integument anterior to the plate, as well a wider, pale sclerite on the posterior edge of segment. The subgenital plate with two large and well-defined lobes, the plate is distinctly bicoloured: lateral sides and lobes dark brown, while the rest of the plate pale brown. The lobes are triangular, not swollen, slightly raised in lateral view, not converging in ventral view and overhang the segment end, the notch between them has wavy sides but the proximal part is triangular. The central plate is smooth, only very slightly and evenly bulging in lateral view. Sternite IX without anterior indentation but the anterior 1/3 is membranous. Paraproct, cercus and epiproct simple. Spermathecal sclerite thin, ring-shaped, with small anterior teeth and converging, longer posterior teeth.

#### Affinities.

The new species is a member of the narrowly defined *hippopus* group (*hippopus* subgroup, sensu, e.g., Ravizza 2002b), and morphologically closest to the West Balkan endemic *L.hippopoides* and the East Balkan endemic *L.pseudohippopus* Raušer, 1965. The male can be distinguished from both on the basis of a nearly fully membranous tergite VII, convergent pair of processes of tergite VIII that delimit the rounded median membranous area, the membranous area triangular lateral to the processes, the small posteromedial sclerite of tergite IX divided into leaf-like portions, and the epiproct sclerotised only at its sides, but with long stalk. Both *L.hippopoides* and *L.pseudohippopus* have a bell-shaped membranous area on tergite VII, the pair of processes not converging on tergite VIII and delimiting a square median membranous area, while the membranous area is rounded laterad to the processes, their epiproct more heavily sclerotised and with a short stalk; the posteromedial sclerite of tergite IX is divided into triangular portions in *L.pseudohippopus*, while the undivided sclerite is large and trapezoidal in *L.hippopoides*. The island endemic *L.pavesii* Vinçon, 2015 from Cephalonia, Greece, resembling *L.hippopoides* but differs by having a straight specillum in lateral view, contrary to the gentle curve for males of all other Balkan species of the species group. The widespread *L.hippopus* Kempny, 1899, co-occurring with the new species, is morphologically less related, and the male can easily be distinguished by its mostly sclerotised tergite VII, robust paired processes of tergite VIII, large and complex posteromedial sclerite of tergite IX, and heavily sclerotised epiproct with a short stalk. The female of the new species distinctly differs from all members of the narrowly defined *hippopus* group by having triangular lobes of the subgenital plate, contrary to the bulging, rounded, square, or finger-like lobes of the other species. The subgenital plate most resembles the not closely related *L.armata* Kempny, 1899, which has a small central lobule between the base of the lobes.

#### Distribution and ecology.

The species was collected at two high elevation localities of the Golija Mts of southwestern Serbia (Fig. 1). The Golija is a south-eastern range of the Dinarids, a mostly volcanic area with a large plateau, its highest peak reaching more than 1800 meters. Most of the specimens were found at the Moravica Stream, 1–2 meters wide, < 0.5 meter deep, running in alder forest (Fig. 6). The other habitat, where only a few specimens were found, is a spring brook that belongs to the watershed of the Ibar River, being rather steep and only 0.5 meter wide with a few centimetres depth, running at the edge of a beech forest. Both streams have stony substrates mixed with gravel and small sandy patches. We visited the localities three times (Murányi and Kovács 2024): the new species was swarming in early May (6.v.2023, leg. L.P. Kolcsár, T. Kovács, D. Murányi), only a few individuals were found in late June (26.vi.2018, leg. P. Juhász, T. Kovács, D. Murányi), while no specimens were present in October (23.x.2023, leg. T. Kovács, D. Murányi, D. Pifkó). At the Moravica Stream, the following species were found to be cohabiting: *Brachypterabulgarica* Raušer, 1962 (adults and larvae, May), *B.seticornis* (Klapálek, 1902) (adults, larvae, and exuviae, May), Capnias.l.viduarilensis Raušer, 1962 (adults, May), *Leuctranigra* (adults, May and June), *L.cingulata* Kempny, 1899 (adults, October), *L.dalmoni* (adults, May) (Fig. 12D, H), *L.prima* Kempny, 1899 (adults, May), L.cf.balcanica Raušer, 1965 (adults, June), *Nemouracinereacinerea* (adults, June), *N.uncinata* Despax, 1934 (adults, May), *N.marginata* (adults, June), an undescribed *Nemoura* species of the *marginata* group (adults, May), an undescribed *Nemoura* related to *N.bulgarica* Raušer, 1962 (adults, June), *Protonemurapraecoxpraecox* (Morton, 1894) (adults, May), *P.intricataintricata* (Ris, 1902) (adults, June), *P.hrabei* Raušer, 1956 (adults, October), *P.nitida* (Pictet, 1836) (adults, October), Perlacf.pallida (larvae, May to October), Isoperlacf.russevi Sowa, 1970 (adults, June), *Chloroperlarussevi* Braasch, 1969 (adults, June) and *Siphonoperlaneglecta* (adults, June). At the spring brook, a different set of species were collected: *Brachypterabulgarica* (adults, May), *Leuctranigra* (adults, May and June), *L.fuscafusca* (Linnaeus, 1758) (adults, October), *L.cingulata* (adults, October), *L.hirsuta* (adults, October), *L.hippopus* (adults, May), *L.dalmoni* (adults, May), *L.prima* (adults, May), L.cf.balcanica (adults, June), *Amphinemurastandfussi* (Ris, 1902) (adults, June), *Protonemurahrabei* (adults, October), an undescribed *Protonemura* species of the *auberti* group (adults in October, larvae in May and June), *Nemouracinereacinerea* (adults, June), *N.marginata* (adults, June), the undescribed *Nemoura* species of the *marginata* group (adults, May), the undescribed *Nemoura* related to *N.bulgarica* (adults, June), Perlacf.pallida (larvae, October), *Isoperlarussevi* (adults, June) and an unidentified *Isoperla* (adult female, June).

#### Etymology.

The name *golija* is derived from the Golija Mountains of Serbia, where the new species was found and is probably restricted to these mountains and the nearby ranges. Used as a noun, gender neutral.

##### ﻿*Leuctraprima* species complex

Within the West Palaearctic, *Leuctra* species are morphologically characterised by having tergal process only on male tergite VIII; closely related autumnal species of this lineage were recently defined as the *L.signifera* group (Reding et al. 2023). A similar, closely related lineage that are emerging during the late winter and spring months can be regarded as the *L.prima* species complex. This species complex consists of three widespread European species (*L.prima*, *L.pseudosignifera* Aubert, 1954, *L.dalmoni*), species restricted to the Alps and the northern Apennines (*L.niveola* Schmid, 1947, *L.helvetica* Aubert, 1956a, *L.ligurica* Aubert, 1962, *L.ravizzai* Ravizza Dematteis & Vinçon, 1994), the Pyrenees (*L.joani* Vinçon & Pardo, 1994), the Transylvanian Alps (*L.transsylvanica* Kis, 1964) and the eastern Balkans (*L.joosti* Braasch, 1970). While redefining the identity of the three widespread species (Vinçon and Murányi 2007), we faced several problems. First, in Western Europe, the Alps, and most of the Carpathians, the morphological distinction of the three species is not problematic; however, we observed notable variability and intermediate (hybrid?) specimens in the Eastern Carpathians and among the sparse Balkan materials. Second, the females of *L.pseudosignifera*, *L.transsylvanica*, and *L.joosti* seemed to be morphologically indistinguishable, and the distinction between the males of *L.transsylvanica* and *L.joosti* proved to be very problematic on the few available specimens. Third, a presumably new species was found among Bosnian specimens, but we postponed its description because only a single male was available. During the last decade, we collected rich materials in the Balkans, and found several populations where the identity cannot be solved on the basis of morphology. Molecular studies are underway, but two distinctive new species can be described on the basis of their morphology and ecology.

### ﻿ Leuctra puskasi

Taxon classificationAnimaliaPlecopteraLeuctridae

Murányi & Kovács sp. nov.

B7518924-CBFD-591F-A01F-C896BA1EA93B

https://zoobank.org/9C2CF7D9-F7F4-469C-AF6D-B78117E2078E

**Figure 8.**
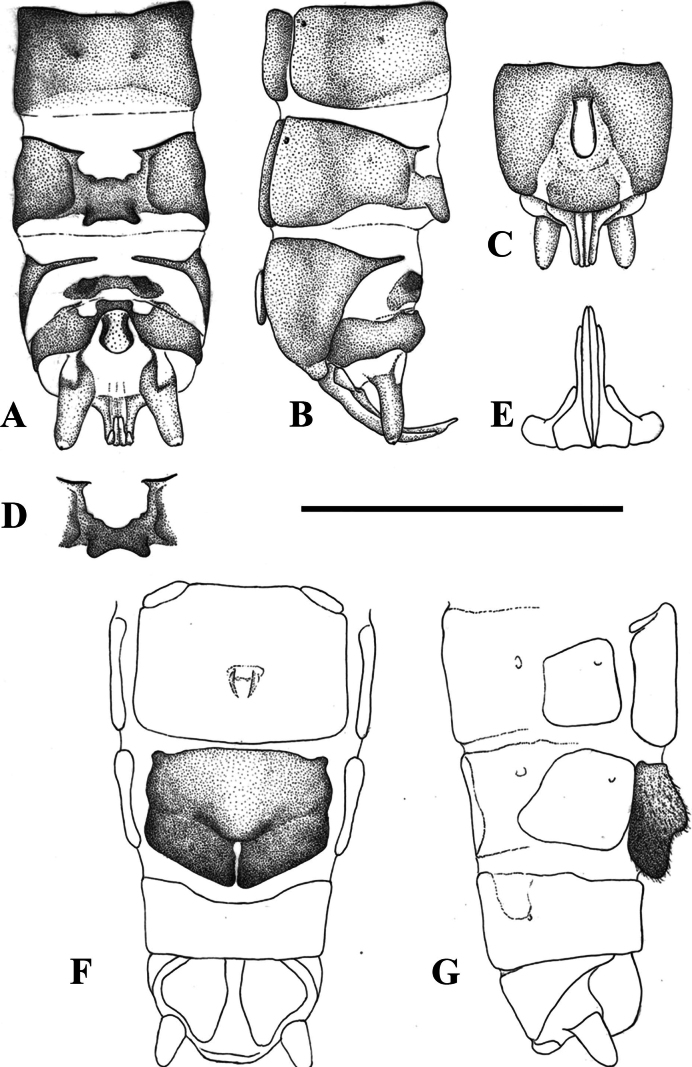
Terminalia of *Leuctrapuskasi* sp. nov. **A** male terminalia, dorsal view **B** same, lateral view **C** same, ventral view **D** variation of male tergite 8, dorsal view **E** male paraprocts and specillae, caudal view **F** female terminalia, ventral view **G** same, lateral view. Setation omitted with the exception of subgenital plate on **G**. Scale bar: 1 mm.

**Figure 9.**
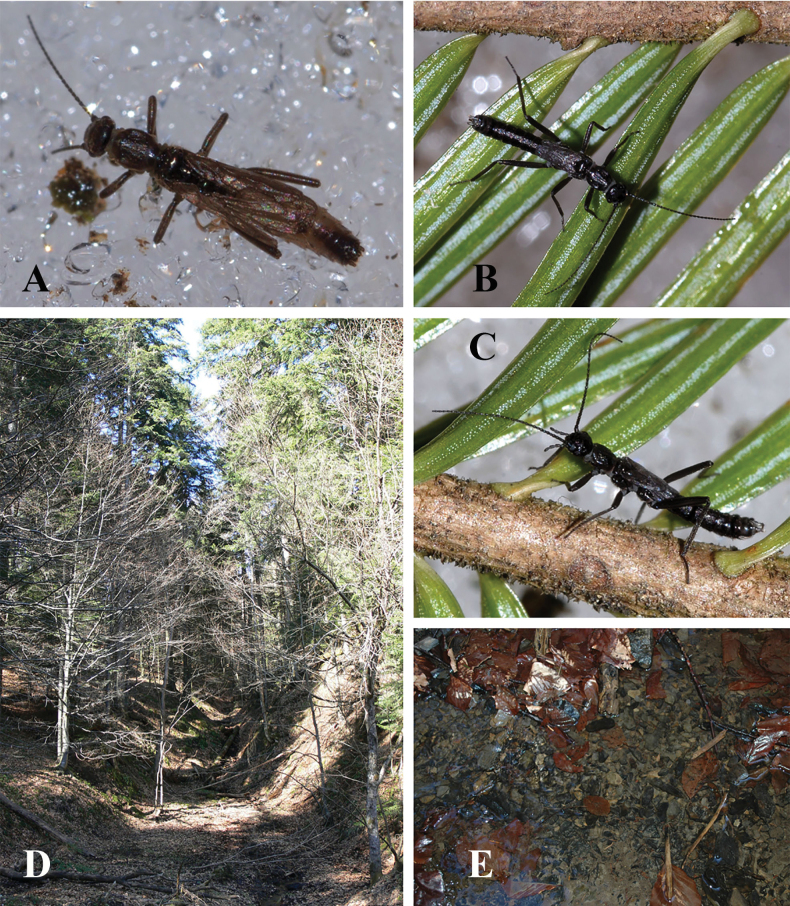
Habitus and habitat of *Leuctrapuskasi* sp. nov. **A** female habitus **B, C** male habitus **D** type locality: forest brook in the Kozara Mts. **E** substrate of the type locality. Photographs **A–C** by Gellért Puskás.

Figs 1 , 8 , 9 , 12A, E

#### Type material.

***Holotype*** male: Bosnia & Herzegovina • Republika Srpska, Kozara Mts, forest brook along the Gornji Podgradci-Kozarac road, 595 m, 45.0414°N, 16.9040°E, 16.iii.2012 (field number: loc.6), leg. T. Kovács, D. Murányi, G. Puskás (HNHM: PLP4185). ***Paratypes***: • same locality and date: 1♂ 1♀ (BYU) • 1♂ 1♀ (CGV) • 10♂ 4♀ (HNHM: PLP3853) • 12♂ 5♀ (MM: 2012-10, PLETYP-36) • 1♂ 1♀ (SMNS).

#### Diagnosis.

Brachypterous in both sexes. Male tergite VII with small membranous portion; tergite VIII with strong posteromedial process that is not erect in side view and bear rounded lobes, membranous area usually shorter than 1/2 of segment length, the posterior margin is nearly straight between the posteromedial process and segment sides; tergite IX with short but wide posteromedial sclerite; tergite X posterior margin with deep but narrow notch; sternite IX bears a vesicle shorter than 1/2 segment length; specillum longer than paraproct, tip subterminally constricted. Female subgenital plate large and trapezoid, incision between the long lobes widening towards the median bulge but not forming a triangular field, median bulge is narrow in ventral view and distinctly raised in lateral view, lacking distinct setation; spermathecal sclerite ring-shaped, with large converging posterior teeth.

#### Description.

Medium sized, slender species, females brachypterous (Fig. 9A), males strongly brachypterous (Fig. 9B, C). Forewing length: holotype 1.4 mm, male paratypes 1.0–1.4 mm, female paratypes 2.6–3.0 mm; body length: holotype 5.4 mm, male paratypes 4.5–5.8 mm, female paratypes 5.5–6.8 mm. Setation generally short and dense. General colour dark brown to blackish. Head and antennae dark brown, palpi brown. Pronotum brown to dark brown, slightly longer than wide and having rounded corners, rugosities distinct. Legs dark brown, tarsi slightly paler. Wings brownish but hyaline, venation brown.

Male abdomen (Figs 8A–E, 12A): Tergite I sclerotised only anteriorly and in lateral stripes, with two medial spots. Transverse row of four pigmented spots distinct on terga II–VII. Terga II and III with medially divided antecosta, tergite II with triangular membranous area anteriorly. Terga IV–VII with entire antecosta and full sclerotised, tergite VII with weakly sclerotised posterior edge and small membranous portion. Tergite VIII: antecosta interrupted by a wide membranous area, each part ends into a small triangular plate; the membranous area is ~ 1/3 segment width, and usually do not reach the 1/2 of the segment length but in some specimens it reach down to 2/3 above the posteromedial process, posterior margin of the membranous portion wavy; the surrounding sclerotisation of the membranous area lighter than the rest of the segment; posteromedial process not darker than the lateral areas of the segment, not erect in side view and not or only slightly overhangs the segment posterior edge, nearly as wide as the membranous area above, bilobed with rounded lobes slightly pointed laterad, the space between the lobes is concave to nearly straight; posterior margin between the posteromedial process and segment sides nearly straight, not indenting nor distinctly lobed. Tergite IX mostly membranous, antecosta interrupted in the medial 1/3 to 1/2 of its length; posteromedial sclerite short but wide, sublateral areas darker, the medial portion usually narrower than the lateral portions. Anterior margin of tergite X bilobed anteriorly, posterior margin with deep notch, not wider than 1/3 tergite width. Epiproct large, posteriorly rounded, sclerotised only at its sides, stalk short. Cercus simple, covered with long setae. Sterna II–VIII simple, sternite IX bears a vesicle shorter than 1/2 segment length, its width more than 1/2 its length; posteromedial 1/2 of the sternum is delimited by a pale and weakly sclerotised area. Paraproct with moderately wide base, abruptly narrowing after basal 1/3, apex gently curved in lateral view and tapering towards a sharp tip. Base of paraproct connected to a subrectangular lateral expansion with an apical, blunt triangular process. Specillum longer than the paraproct, gently curved in lateral view and ending in a subterminally constricted, sharp tip.

Female abdomen (Figs 8F, G, 12E): Terga I–VIII with transverse row of four pigmented spots clearly visible; terga I–IV mostly membranous but with lateral sclerites, terga V–VIII with medial sclerite as well; medial sclerite is a longitudinal stripe on tergite V, gradually widens on the following segments and as wide as 1/2 segment width on tergite VIII; terga IX–X fully sclerotised. Sterna I–VII simple, sterna II–VII with one subrectangular median sclerite and two small anterior sclerites that are fused with the median sclerite on sternite VII. Subgenital plate of sternite VIII large and trapezoid, not fused with other sclerites. It has two large and wide lobes, slightly darker brown than the rest of the plate; posterior margin convex, incision between the lobes is a membranous strip, widening towards the median bulge near the centre of the plate but not forming a triangular field; median bulge is narrow in ventral view, distinctly raised, nose shaped to rounded in lateral view, lacking distinct setation. Sternite IX with wide but shallow anterior indentation. Paraproct, cercus and epiproct simple. Spermathecal sclerite ring-shaped, with small anterior teeth and large, converging the posterior teeth.

#### Affinities.

**Figure 10.**
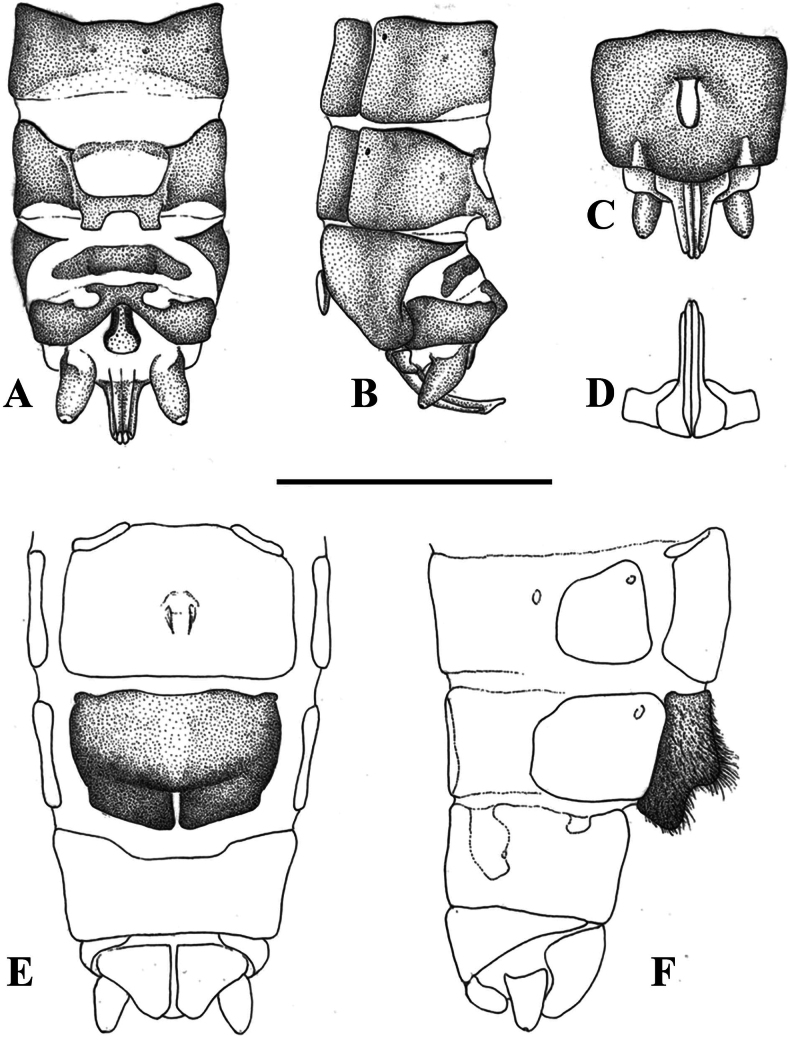
Terminalia of *Leuctravisitor* sp. nov. **A** male terminalia, dorsal view **B** same, lateral view **C** same, ventral view **D** male paraprocts and specillae, caudal view **E** female terminalia, ventral view **F** same, lateral view. Setation omitted with the exception of subgenital plate on **F**. Scale bar: 1 mm.

Both sexes are morphologically the closest to *L.dalmoni* (compare with Fig. 12C, D). Males can be distinguished on the basis of the following characters: posteromedial process of tergite VIII stronger, its lobes are not teeth-like, and preceded by a more shallow membranous area than of those of *L.dalmoni*; the posterior margin of tergite VIII is nearly straight between the posteromedial process and segment sides, while it is clearly indented in a rounded membranous field in *L.dalmoni*; the posterior indentation of tergite X is a deep notch that is not wider than 1/3 tergite width, while that is usually wider than 1/2 tergite width in *L.dalmoni*; the ventral vesicle of sternite IX is shorter than 1/2 segment length, while that is clearly longer than 1/2 segment length in *L.dalmoni*. Females are nearly identical (compare with Fig. 12G, H), but variability might be high; however, in the available females, the incision between the lobes of the subgenital plate widens towards the median bulge, while it is only a narrow strip in *L.dalmoni*, and the median bulge is more robust in lateral view. The males of the new species can be easily distinguished from the other regional early spring species of the *prima* species complex: *L.pseudosignifera* is characterised by a pair of rounded sclerotised expansions, located between the posteromedial process and segment sides of tergite VIII (Fig. 12K); the posteromedial process of tergite VIII is erect in lateral view in case of *L.prima.* Furthermore, this species has a divided posteromedial sclerite on tergite IX (Fig. 12I); *L.visitor* has a crossband on the membranous area of tergite VIII, the membranous area is deeper, and the posteromedial process has stronger, angular teeth; additionally, the posterior notch of tergite X is a wide V-shape (Fig. 12B). The females are also easily distinguishable: *L.pseudosignifera* is characterised by a triangular membranous field at the end of the incision between the lobes of the subgenital plate (Fig. 12L); the median bulge of the subgenital plate is much wider in *L.prima*, and the lobes of the subgenital plate are shorter (Fig. 12J); *L.visitor* has a wide median bulge of the subgenital plate, and the plate has distinctive, erect setation in lateral view (Fig. 10F). In addition, all the other species are macropterous.

#### Distribution and ecology.

The species was collected at a single forest brook in the Kozara Mts of northwestern Bosnia & Herzegovina (Fig. 1). The Kozara is of low elevation, a northern foothill of the Dinarids, rather separated from the higher ranges and also stands far from the Slavonian mountains, separated by the plain of the Sava River. The habitat is a slowly running small brook in mixed forest dominated by fir and beech, which probably often runs dry in summer (Fig. 9D). It has a stony substrate mixed with mud and plenty of dead wood and fallen leaves (Fig. 9E). This habitat type is very different from those of the closely related *L.dalmoni*, and probably unsuitable even for the more eurytopic *L.prima*. No other member of the *Leuctraprima* species complex was found in the Kozara, despite the fact that some other habitats seemed suitable at least for *L.prima*. The type specimens were the only adult stoneflies found there (in partial snow cover), but last instar larvae of *Leuctranigra*, *Nemourasciurus* Aubert, 1949, and a *Nemouramarginata* group member were found in the brook. We visited the locality twice more, once in May (24.v.2012, leg. T. Kovács, G. Puskás) and once in November (7.xi.2012, leg. T. Kovács, G. Magos). In May, adults of *Leuctranigra*, single females of a *N.marginata* group member and of *Siphonoperlatorrentium* (Pictet, 1841) were collected. In November, no stoneflies but only autumnal Trichoptera were collected (Oláh and Kovács 2012a; Oláh et al. 2012): *Chaetopteryxgonospina*, *C.papukensis*.

#### Etymology.

We dedicate this species to our friend and orthopterologist colleague, Gellért Puskás, with whom we made several collecting expeditions in the Balkans, and who regularly provides us stoneflies from his travels. Used as a noun, gender masculine.

### ﻿ Leuctra visitor sp. nov.

Taxon classificationAnimaliaPlecopteraLeuctridae

83CD7F7B-7781-58F0-B107-C65E2DED1906

https://zoobank.org/6E2C2B5D-5228-4A6A-B501-C2FB65A162EC

**Figure 11.**
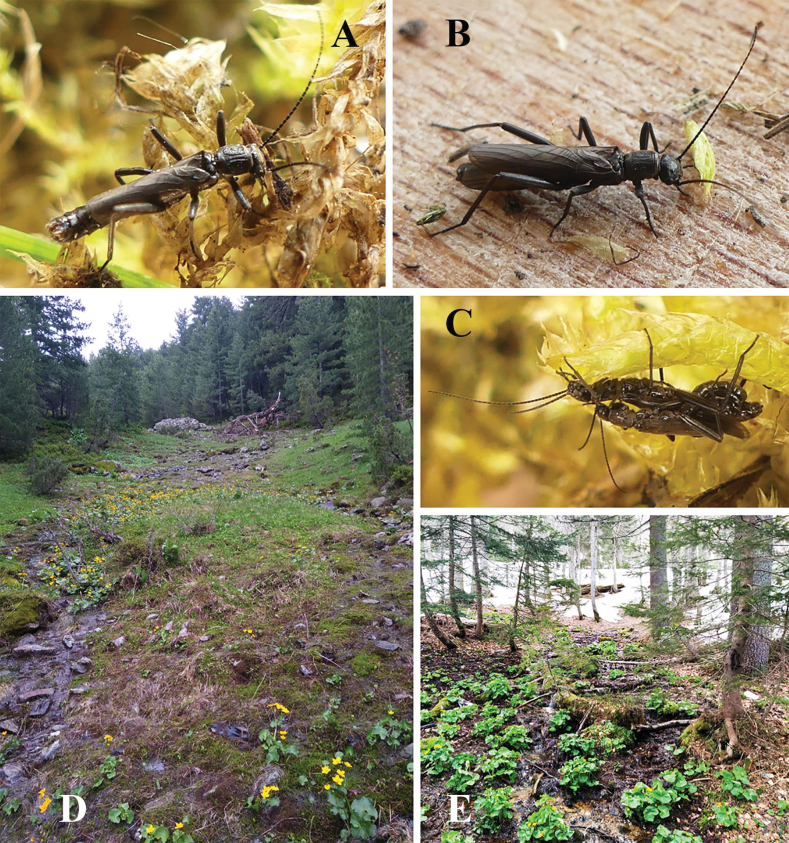
Habitus and habitat of *Leuctravisitor* sp. nov. **A** male habitus **B** female habitus **C** copula **D** partly open brook at 1845 m in the Visitor Mts. **E** type locality: forest springs and their outlet at 1730 m in the Visitor Mts.

**Figure 12.**
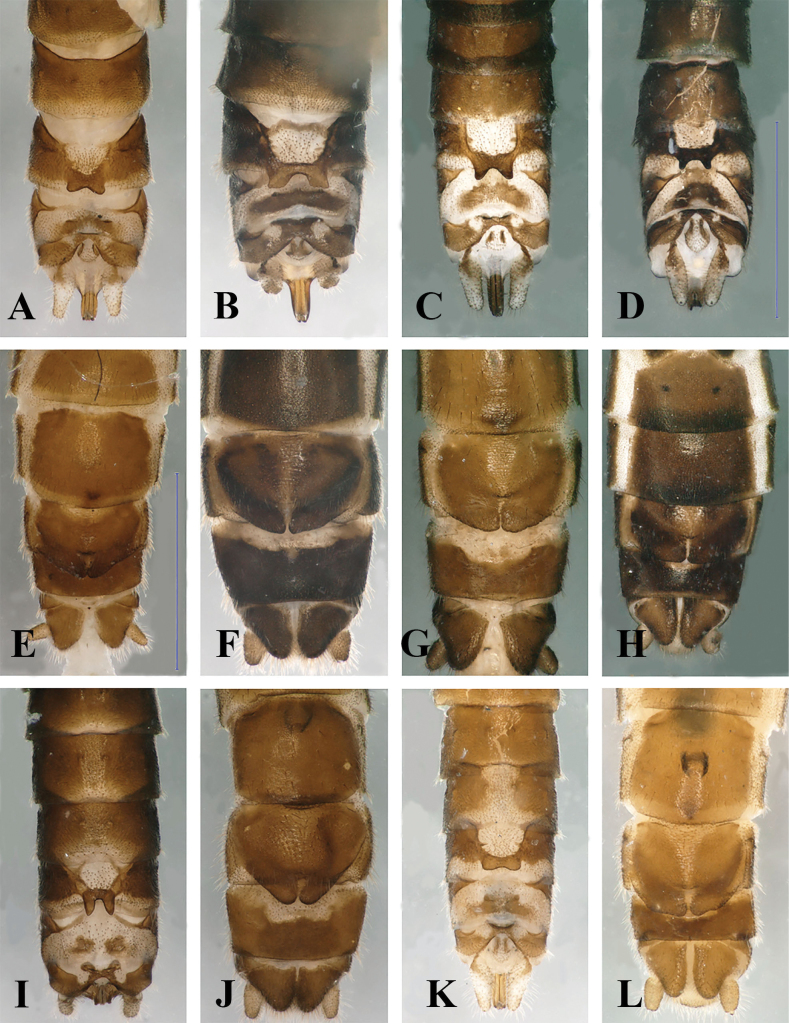
Terminalia of five Balkan members of the *Leuctraprima* group **A–D, I, K** male terminalia, dorsal view **E–H, J, L** female terminalia, ventral view **A, E***L.puskasi* sp. nov., Kozara Mts, Bosnia & Herzegovina **B, F***L.visitor* sp. nov., Visitor Mts, Montenegro **C, G***L.dalmoni* Vinçon & Murányi, 2007, Visitor Mts, Montenegro **D, H***L.dalmoni*, Golija Mts, Serbia **I–J***L.prima* Kempny, 1899, Börzsöny Mts, Hungary **K–L***L.pseudosignifera* Aubert, 1954, Poljana Mts, Slovakia. Scale bars: 1 mm (**D** for **A–D, I, K; E** for **E–H, J, L**).

Figs 1 , 10 , 11 , 12B, F

#### Type material.

***Holotype*** male: Montenegro: • Plav municipality, Visitor Mts, forest springs and their outlet along the Katun road, 1730 m, 42.6308°N, 19.8358°E, 5.v.2023 (field number: loc.13), leg. T. Kovács, D. Murányi (MM: 2023-49, PLETYP-37). ***Paratypes***: • same locality and date: 1♂ 1♀ (BYU) • 1♂ 1♀ (CGV) • 11♂ 8♀ (3 pairs in copula) (EKCU: PLP5706) • 1♂ 2♀ (MM: 2023-49, PLETYP-38) • 1♂ 1♀ (SMNS) • Plav municipality, Visitor Mts, partly open brook and spring along the Katun road, 1845 m, 42.6185°N, 19.8378°E, 11.vi.2022 (loc.24), leg. A. Hunyadi, T. Kovács, D. Murányi, P. Olajos: 5♂ 3♀ (EKCU: PLP5584) • 8♂ 9♀ (1 pair in copula) (MM: 2022-90, PLETYP-39) • Plav municipality, Visitor Mts, forest streams and brooks along the Katun road, 1515 m, 42.6294°N, 19.8442°E, 11.vi.2022 (loc.22), leg. A. Hunyadi, T. Kovács, D. Murányi, P. Olajos: 1♀ (EKCU: PLP5572) • same locality, 5.v.2023 (loc.12), leg. L.P. Kolcsár, T. Kovács, D. Murányi: 8♂ 7♀ (2 pairs in copula) (EKCU: PLP5701) • 6♂ 8♀ (1 pair in copula) (MM: 2023-48, PLETYP-40).

#### Diagnosis.

Brachypterous in both sexes. Male tergite VII with small membranous portion; tergite VIII with strong posteromedial process that is not erect in side view and bear angular teeth, membranous area longer than 1/2 segment length with a crossband, the posterior margin indented in a rounded membranous field between the posteromedial process and segment sides; tergite IX with short but wide posteromedial sclerite; tergite X posterior margin with a wide V-shaped notch; sternite IX bears a vesicle much shorter than 1/2 segment length; specillum slightly longer than paraproct, tip blunt behind subterminal constriction. Female subgenital plate large, trapezoid, with an incision between the long lobes slightly widening towards the median bulge but not forming a triangular field; median bulge is wide in ventral view and distinctly raised in lateral view, with distinct, erect setation; spermathecal sclerite ring-shaped, with large and converging posterior teeth.

#### Description.

Medium sized, robust species, both sexes brachypterous (Fig. 11A–C). Forewing length: holotype 3.2 mm, male paratypes 3.0–3.8 mm, female paratypes 4.2–5.2 mm; body length: holotype 6.2 mm, male paratypes 5.4–7.0 mm, female paratypes 6.8–9.0 mm. Setation generally short and dense. General colour dark brown to blackish. Head, antennae, and palpi dark brown. Pronotum dark brown, as wide as long and having rounded corners, rugosities distinct. Legs brown to dark brown. Wings brownish but hyaline, venation brown.

Male abdomen (Figs 10A–D, 12B): Tergite I membranous in its whole medial portion, tergite II membranous anteromedially behind antecosta. Transverse row of four pigmented spots distinct on terga I–VII. Terga II and III with medially divided antecosta, terga IV–VII with entire antecosta and full sclerotised, tergite VII with small posterior membranous portion. Tergite VIII: antecosta interrupted by a wide membranous area, each part ends in a small triangular plate, and the membranous area between them is bridged by a weakly sclerotised crossband; width of the membranous area is less than 1/2 the segment width, and reaches below 1/2 the segment length above the posteromedial process, posterior margin of the membranous portion nearly straight; the surrounding sclerotisation of the membranous area paler than remainder of the segment; posteromedial process paler than the lateral areas of the segment, not erect in side view and slightly overhangs the segment posterior edge, as wide as the membranous area above, bilobed with angular teeth slightly pointed laterad, the space between the teeth is deeply indented and concave; posterior margin between the posteromedial process and segment sides indented in a shallow, rounded membranous field but not lobed. Tergite IX mostly membranous, antecosta interrupted in the medial 1/2 of its length; posteromedial sclerite short but wide, medial areas usually slightly darker, the medial portion usually not narrowed. Anterior margin of tergite X bilobed anteriorly, posterior margin with deep, V-shaped notch, 1/2 as wide as tergite width. Epiproct large, posteriorly rounded, sclerotised only at its sides, stalk relatively long. Cercus short, covered with long setae. Sterna II–VIII simple, sternite IX bears a vesicle much shorter than 1/2 segment length, its width more than 1/2 its length; the area surrounding the vesicle is pale, posterior edge with two pale and weakly sclerotised areas. Paraproct with moderately wide base, abruptly narrowed after basal 1/3, apex gently curved in lateral view and tapering towards a sharp tip. Base of paraproct connected to a subrectangular lateral expansion with an apical, short, blunt triangular process. Specillum slightly longer than the paraproct, gently curved in lateral view and ending in a subterminally constricted, blunt tip.

Female abdomen (Figs 10E, F, 12F): Terga I–VIII with transverse row of four pigmented spots clearly visible; terga I–VIII mostly membranous but with lateral sclerites, terga II, III, and VI–VIII, sometimes also tergite V, bear a patch-like medial sclerite, largest on tergite VIII; tergite IX membranous only anterolaterally, tergite X fully sclerotised. Sterna I–VII simple, sterna II–VII with one subrectangular median sclerite and two small anterior sclerites that are fused with the median sclerite on sternite VII. Subgenital plate of sternite VIII large and trapezoid, not fused with other sclerites. It has two large wide lobes, slightly darker brown than remainder of the plate; posterior margin convex, incision between the lobes is a membranous strip, slightly widened towards the median bulge near the centre of the plate but not forming a triangular field; median bulge is wide in ventral view, distinctly raised, nose-shaped in lateral view, with distinct erect setation. Sternite IX with wide but shallow anterior indentation. Paraproct, cercus, and epiproct simple. Spermathecal sclerite ring-shaped, with small anterior teeth and large, converging posterior teeth.

#### Affinities.

The males are morphologically closest to *L.dalmoni* (compare with Fig. 12C, D) while the female is closest to *L.prima* (compare with Fig. 12J). Males can be distinguished from *L.dalmoni* on the basis of the following characters: a weak crossband on the membranous area of tergite VIII lacking in *L.dalmoni*, angular teeth of the posteromedial process of tergite VIII stronger than those of *L.dalmoni*; the posterior indentation of tergite X is V-shaped, while it is rounded in *L.dalmoni*; the ventral vesicle of sternite IX is shorter than 1/2 the segment length, while clearly longer than 1/2 the segment length in *L.dalmoni*. The males of the new species can be easily distinguished from *L.prima* and *L.pseudosignifera*: *L.pseudosignifera* is characterised by a pair of rounded sclerotised expansions, located between the posteromedial process and segment sides of tergite VIII (Fig. 12K); the posteromedial process of tergite VIII is erect in lateral view in *L.prima*; furthermore, this species has a divided posteromedial sclerite on tergite IX (Fig. 12I). Females can be distinguished from *L.prima* on the basis of the distinct, erect hairs of the subgenital plate as seen in lateral view, and the nose-shape of the bulge of the subgenital plate; the bulge is rounded in *L.prima* and the subgenital plate lacks distinct setation. The wide bulge of the subgenital plate (in ventral view), and the erect hairs (in lateral view) distinguish the new species from *L.dalmoni* and *L.pseudosignifera*; furthermore, *L.pseudosignifera* is characterised by a triangular membranous field at the end of the incision between the lobes of the subgenital plate (Fig. 12L). Distinction of both sexes from *L.puskasi* was described in detail above.

#### Distribution and ecology.

The species was collected at three high-elevation brooks and spring outlets in the Visitor Mts of eastern Montenegro (Fig. 1). The Visitor is a mostly volcanic range of small area, with its highest peak reaching more than 2200 meters, surrounded by the notably higher limestone chains of the Prokletije Mts. The Visitor receives high precipitation and is covered with dense forest, resulting in a rich pattern of different waterflows. The streams where the new species was found are running in mixed forest or partly open meadows, the lentic parts usually with dense *Caltha* growth (Fig. 11D, E). The brooks have variable flow rates but are permanent streams, the substrate is stony mixed with mud and plenty of dead wood. The specimens were emerging together with *L.dalmoni* (Fig. 12C, G) and both species were present with many mating pairs in May, but we found no interbreeding couples (Fig. 11C). We visited the area three times: the new species was more numerous than *L.dalmoni* in early May 2023, both scarce in early June 2022, and both absent by late June 2023. In May, the upper site (1845 m) was not available, and even the lowest site (1515 m) was partly covered by snow, but all were snow-free by late June. We found a rich accompanying stonefly fauna at the lowest site: *Brachypterahelenica* (adults and matured larvae, early June), *B.graeca* Berthélemy, 1971 (adults, late June), *B.seticornis* (adults, early June), Capnias. l.viduarilensis (adults, May and early June), *Leuctranigra* (adults, May and June), *L.dalmoni* (adults, May and early June), *L.metsovonica* Aubert, 1956b (adults, early June), Protonemuracf.auberti Illies, 1954b (adults and matured larvae, early June), *Nemurellapictetii* (adults, May and June), *Nemouracinereacinerea* (adults, early June), *Nemouramarginata* (adults, May and early June), a yet unnamed *Nemoura* of the *marginata* group (adults, May and early June), *N.uncinata* Despax, 1934 (adults, May and early June), *Arcynopteryxdichroa* (McLachlan, 1872) (adults and matured larvae, June), a yet unnamed *Isoperla* related to *I.breviptera* Ikonomov, 1980 (adults and matured larvae, June), I.cf.tripartita Illies, 1954a (larvae, June), and unidentified larvae of *Siphonoperla* and *Chloroperla* (early June). Some of these were probably not developing in the same stream section since the site is placed at a junction of three different brooks. The type locality (sampled only in May) was inhabited by the new species, *L.nigra* (adults), *L.dalmoni* (adults), the unnamed *Nemoura* (adults and putative larvae), an unidentified *Protonemura* (larvae), and the unnamed *Isoperla* (larvae). At the highest site (sampled only in June), the new species was found together with *Leuctranigra* (adults), *L.dalmoni* (adults), the unnamed *Nemoura* (adults), *Nemurellapictetii* (adults), *Arcynopteryxdichroa* (larvae), and the unnamed *Isoperla* (adults). Capnias. l.viduarilensis, *Leuctradalmoni*, and *L.metsovonica* are new records for Montenegro.

#### Etymology.

The name *visitor* is derived from the Visitor Mountains of Montenegro, where the new species was found and probably restricted to the high elevations of the range. Used as a noun, gender neutral.

## ﻿Discussion

Four new, presumably microendemic, species are described from different regions of the western Balkans. *Leuctraenigma* is a morphologically isolated member of the *L.hippopus* group, found in a single mountain stream in central Albania. *Leuctragolija* is closely related to the Balkan endemics *L.hippopoides* and *L.pseudohippopus*, and was found in the higher region of the Golija Mts in southwestern Serbia. Two brachypterous members of the *L.prima* species complex were described from the Kozara Mts of Bosnia & Herzegovina and the Visitor Mts of Montenegro. Molecular studies are underway to clarify the composition of this species complex in the Balkans, but these two species are morphologically distinctive and their ecology supports their specific status: *L.puskasi* is the only member of the complex in the Kozara, while *L.visitor* coexists with *L.dalmoni* in the Visitor but the two do not interbreed.

To date, there are 48 species and one subspecies of *Leuctra* reported from the Balkans, including the Aegean Isles. More than half of these taxa are endemic to the region (27 taxa), and their distributions and emergence periods are given in Table 1. Only ten of them have a wider distribution within the Balkan mainland. A further six are endemic to one or more of the Greek islands, and eleven (including the four new species described herein) are restricted to a single mountain chain. The mountain endemics are mostly of reduced or lost flight capability (three apterous, three micropterous to strongly brachypterous, two brachypterous); all others, including the island endemics, are macropterous.

**Table 1.** Balkan endemic *Leuctra*, with their emergence periods and distributions.

Species/subspecies	Emergence period	Distribution
***fusca* group**		
*L.aegaeica* Pardo & Zwick, 1993	X–III	Andros, Euboea, Naxos (Greece)
*L.candiae* Zwick, 1978	IX–IV	Crete (Greece)
*L.cretica* Zwick, 1978	X–V	Crete (Greece)
*L.graeca* Zwick, 1978	IX–X	Albania, Greece, Montenegro, North Macedonia
*L.kykladica* Pardo & Zwick, 1993	X	Naxos (Greece)
*L.minoica* Pardo & Zwick, 1993	XII–II	Crete (Greece)
*L.moreae* Zwick, 1978	X–V	Greece
*L.mortonifeheri* Murányi, 2007	IX–X	Albania, Bulgaria
***hippopus* group**		
*L.enigma* sp. nov.	X	Çermenikë Mts (Albania)
*L.golija* sp. nov.	V–VI	Golija Mts (Serbia)
*L.hippopoides* Kaćanski & Zwick, 1970	IV–V	Albania, Bosnia & Herzegovina, Greece *, Montenegro, Serbia
*L.pavesii* Vinçon, 2015	III	Cephalonia (Greece)
*L.pseudohippopus* Raušer, 1965	V–VII	Bulgaria, North Macedonia, Serbia
***prima/signifera* group**		
*L.hansmalickyi* Graf, 2010	VI	Rila Mts (Bulgaria)
*L.helenae* Braasch, 1972	IX	Stara Planina (Bulgaria)
*L.jahorinensis* Kaćanski, 1972	X	Jahorina Mts (Bosnia & Herzegovina) **
*L.joosti* Braasch, 1970	IV–VI	Bulgaria, Turkey (European part)
*L.kumanskii* Braasch & Joost, 1977	X	Pirin Mts (Bulgaria)
*L.olympia* Aubert, 1956b	IV–V	Bosnia & Herzegovina, Greece, Montenegro, North Macedonia, Serbia
*L.malcor* Murányi, 2007	X	Prokletije Mts (Albania)
*L.marani* Raušer, 1965	IV–VIII	Bulgaria, Greece
*L.papukensis* Reding, Vinçon & Graf, 2023	X	Papuk Mts (Croatia)
*L.puskasi* sp. nov.	III	Kozara (Bosnia & Herzegovina)
*L.visitor* sp. nov.	V–VI	Visitor Mts (Montenegro)
***inermis* group**		
*L.aptera* Kaćanski & Zwick, 1970	IV	Sutjeska (Bosnia & Herzegovina)
*L.balcanica* Raušer, 1965	V–VII	Bulgaria
*L.metsovonica* Aubert, 1956b	IV–VIII	Albania, Greece, Montenegro, North Macedonia ***

* Greek specimens of *L.hippopus* were reported to have intermediate characters towards *L.hippopoides* by Zwick (1978). ** records from Montenegro (Murányi 2011) needs confirmation. *** records from Italy (Pardo and Zwick 1993) needs confirmation.

## Supplementary Material

XML Treatment for Leuctra enigma

XML Treatment for Leuctra golija

XML Treatment for Leuctra puskasi

XML Treatment for Leuctra visitor

## ﻿Additional information

### Author ORCIDs

Dávid Murányi https://orcid.org/0000-0002-3907-5590

Tibor Kovács https://orcid.org/0000-0003-4577-0394

### Data availability

All of the data that support the findings of this study are available in the main text.
